# Analysis of possible baseline and treatment-course factors associated with non-remission in patients with Crohn’s disease treated with ustekinumab: a retrospective real-life analysis

**DOI:** 10.1007/s43440-026-00847-5

**Published:** 2026-03-24

**Authors:** Antonio Tursi, Raffaele Pellegrino, Giammarco Mocci, Walter Elisei, Franco Scaldaferri, Edoardo V. Savarino, Giovanni Maconi, Giorgia Bodini, Alfredo Papa, Antonietta Gerarda Gravina

**Affiliations:** 1 Territorial Gastroenterology Service, ASL BAT, Via Torino, 49, 76123 Andria, BT Italy; 2 Department of Medical and Surgical Sciences, School of Medicine, Catholic University, 00168 Rome, Italy; 3 Department of Precision Medicine, Hepatogastroenterology and Digestive Endoscopy Unit, University of Campania “Luigi Vanvitelli”, 80138 Naples, Italy; 4 Division of Gastroenterology, AORN “Brotzu” Hospital, 09124 Cagliari, Italy; 5 Division of Gastroenterology, A.O. “S. Camillo-Folanini”, 00152 Rome, Italy; 6 Digestive Diseases Centre (CEMAD), Department of Medical and Surgical Sciences, Policlinico Universitario “A. Gemelli” Foundation, IRCCS, Largo Francesco Vito, 1, 00168 Rome, Italy; 7 School of Medicine, Catholic University, 00168 Rome, Italy; 8 Gastroenterology Unit, Azienda Ospedale-Università di Padova (AOUP), 35100 Padua, Italy; 9 Gastroenterology Unit, Department of Biomedical and Clinical Sciences, “L. Sacco” University Hospital, 20100 Milan, Italy; 10 Department of Internal Medicine and Medical Specialities, Division of Gastroenterology, IRCCS “San Martino” Hospital, University of Genoa, 86100 Genoa, Italy

**Keywords:** Crohn’s disease, Ustekinumab, Clinical remission, Predictive factors, Faecal calprotectin, Appendectomy, 5-aminosalicylic acid, Steroids

## Abstract

**Background:**

Factors predicting persistent failure to achieve long-term clinical remission with ustekinumab (UST) in Crohn’s disease (CD) are poorly defined, limiting early identification of patients unlikely to benefit from continuation of an ineffective treatment.

**Methods:**

A multicentre retrospective registry was established comprising adult patients with CD treated with UST (induction as per guidelines and maintenance 90 mg every 8 weeks), including clinico-demographic variables and disease activity parameters. These variables were retrospectively collected at baseline (T_0_) and at 2 (T_1_), 3 (T_2_), 6 (T_3_) and 12 (T_4_) months. The primary endpoint was the identification of factors associated with failure to achieve clinical remission (defined as an HBI ≥ 5) at T_3_, with the same assessment performed at T_4_ as a secondary endpoint.

**Results:**

A total of 254 patients were included in the T_3_ analysis and 143 in the T_4_ analysis. At T_3_, lack of remission was associated with prior exposure to 5-ASA (aOR 2.704, 95%CI 1.423–5.137), appendectomy (aOR 1.965, 95%CI 1.008–3.829) and CD diagnosis before the age of 40 (aOR 2.03, 95%CI 1.072–3.834). At T_4_, appendectomy confirmed the association (aOR 2.938, 95%CI 1.212–7.124). A baseline FC cut-off ≥ 250 µg/g predicted lack of remission at six months (aOR 3.57, 95%CI 1.686–7.561).

**Conclusion:**

Several anamnestic and on-treatment variables warrant evaluation in this setting, pending confirmation in larger datasets.

**Supplementary Information:**

The online version contains supplementary material available at 10.1007/s43440-026-00847-5.

## Introduction

Ustekinumab (UST), a monoclonal antibody blocking the p40 subunit of the interleukin (IL) 12/23 [1], was granted marketing authorisation in November 2016 by the European Medicines Agency for the treatment of moderate-to-severe Crohn’s disease (CD) in adult patients with inadequate response, loss of response, or intolerance to either conventional therapies or biologics [2].

This drug, which is also currently approved for the treatment of psoriasis and ulcerative colitis [3, 4], has demonstrated significant efficacy not only in controlled trials [5, 6] but also in real-world studies [7–10]. We recently found that UST can achieve and maintain remission for up to 24 months in the majority of patients [8]. Moreover, about one-fourth of patients who reach clinical response, but not clinical remission, can achieve remission over 12 months of UST treatment [8]. However, a small percentage of these patients do not benefit from UST treatment, even when the treatment is extended up to 24 months [8].

It is therefore important to identify the characteristics of these patients, as this could help us determine who is likely to benefit from UST treatment with a high probability of therapeutic success and who is not. Several recent studies tried to identify prognostic factor of response to UST. A short disease duration (≤ 2 years) and higher baseline serum albumin level [11], as well as less exposure to previous biologics [12], have been identified as positive prognostic factors of response to UST. Less is currently known about the negative prognostic factors of response to UST. Higher C-reactive protein (CRP) at baseline [9], older age, higher body mass index, fecal calprotectin levels at baseline, the number of concomitant steroid courses [12] or smoking habits [13] have been identified as negative prognostic factor of response to UST. However, the majority of these data arise from short-term follow-up or from small sample size. The present study aimed to identify prognostic factors of non-remission that may influence failure to achieve this outcome under UST, even in the long term.

## Materials and methods

### Study design and setting, and inclusion criteria

This study has an observational, retrospective, multicentre, real-life design, collecting data from Italian IBD centres that administer advanced medical therapies to outpatients with CD.

Patients were included if they had an endoscopic and histological diagnosis of CD and had received an indication for UST treatment in accordance with the summary of product characteristics (including the management and the prophylaxis of post-operative CD recurrence).

Only adult patients (i.e., at least 18 years of age at the start of UST therapy) managed on an outpatient basis were included, provided that none of the following applied: doubtful diagnosis of CD; clinically significant intestinal strictures (with pre-stricture loop dilatation or clinically active strictures with subocclusive or occlusive symptoms); patients with complications (e.g., fistulas or abscesses) that could only be managed surgically; patients with permanent stomas; patients who had undergone extensive bowel resections (i.e., ≥ two bowel surgical resections and/or ≥ 50 cm ileal resection) leading to short bowel syndrome [14]; patients with intestinal failure; or patients requiring dual therapy (i.e., therapy with two biologics or with one biologic and a small molecule) because of disease complexity and/or management of multiple extra-intestinal manifestations.

As the study was observational and retrospective, it did not involve additional interventions or hospital visits beyond those that would have been part of the routine clinical management of these patients. The study was therefore conducted in accordance with clinical practice guidelines and the principles of the Declaration of Helsinki. All patients provided written informed consent before undergoing endoscopy and UST treatment (as per the standard clinical practice of the participating centres). The reference centre (Brotzu Hospital, Cagliari, Italy, protocol no. PG/2020/9414, dated 29 April 2020) obtained ethics committee approval for this retrospective study, which the other participating centres accepted.

The centres used a single shared database to enter retrospective data for all included patients, following the explicitly agreed inclusion criteria.

### UST treatment

Each patient included in the retrospective analysis had been treated with UST (Stelara^®^, Janssen Biotech, Inc., Beerse, Belgium) in accordance with Annex I [15] filed with the European Medicines Agency, with induction by means of a single intravenous weight-based dose using 130 mg vials. Specifically, for a body weight of ≤ 55 kg, a dose of 260 mg was administered. For a body weight of > 55 kg to ≤ 85 kg, a dose of 390 mg was used, and for a body weight of > 85 kg, a dose of 520 mg was administered. No adjustments were made for elderly patients, as per the Summary of Product Characteristics. Maintenance therapy, in line with real-world practice at the centres and in compliance with the aforementioned Annex I, was administered as 90 mg subcutaneously every 56 days.

### Collected variables and time points

The variables considered in the present study were collected at baseline (T_0_) and at 2 (T_1_), 3 (T_2_), 6 (T_3_), and 12 (T_4_) months following treatment. Indeed, given that this is a real-life study, we acknowledged a tolerance of ± 10 days with respect to the predefined timeline.

Several variables were recorded, specifically clinical-demographic variables (e.g., age, sex, history of appendectomy, and duration of CD) and CD-specific variables (such as the Montreal classification [16], prior surgery, previous treatments, and ongoing treatments). In addition, during the retrospective follow-up, data on disease activity were collected. Clinical disease activity was assessed with the Harvey–Bradshaw Index (HBI) [17], whereas biochemical activity was assessed using the Erythrocyte Sedimentation Rate (ESR), C-Reactive Protein (CRP), and Faecal Calprotectin (FC).

Finally, endoscopic disease activity, where data were available, was assessed with the Simple Endoscopic Score for Crohn’s Disease (SES-CD) [18] in patients without prior ileocaecal resection and ileocolic anastomosis; in the latter cases, the Rutgeerts score [19] was applied.

Variables collected after UST initiation (i.e., T_1_–T_2_) were analysed as early on-treatment indicators, reflecting disease activity and treatment trajectory rather than baseline predictors.

### Study endpoints

The primary endpoint of this study was to identify variables (among those collected) associated with failure to achieve clinical remission (defined as an HBI ≥ 5) at 6 months from treatment initiation (T_3_). In addition, the study included several secondary endpoints. The first secondary endpoint was to conduct the same assessment as for the primary endpoint, but at 12 months from treatment initiation (T_4_). Additionally, a secondary endpoint was to assess the correlation between continuous variables and the HBI at both T_3_ and T_4_. Finally, a secondary endpoint was to assess any variables associated with failure to achieve steroid-free clinical remission (defined as clinical remission according to the same criteria previously specified, but without the use of steroids at the given time point) at T_3_ and T_4_.

### Statistical analysis

Descriptive statistics were employed for data presentation, with continuous variables expressed as median (interquartile range) based on the normality of the data, which was assessed using the Kolmogorov-Smirnov or Shapiro-Wilk tests. Categorical and ordinal variables were presented as frequencies, reporting the absolute number and the percentage of the total. Qualitative non-continuous variables were compared using the Chi-square (χ²) test or Fisher’s exact test, as appropriate. For comparisons involving ordinal and continuous variables, the Mann-Whitney U test or the Kruskal-Wallis test was applied, depending on the degrees of freedom of the grouping variable. Bonferroni correction was applied for multiple comparisons. When correlations were performed, these were conducted using Spearman’s test, with the corresponding Spearman’s coefficient (ρ) also reported. Binary logistic regressions were estimated for each model with all pre-specified covariates entered using a forced-entry approach. The dependent variable was failure to achieve clinical remission (at both study endpoint time points). Missing data for any parameters included as covariates were handled by listwise deletion (complete-case analysis), excluding subjects with at least one missing value in any of the model variables, under the assumption of a missing-at-random mechanism conditional on the observed covariates. The models were evaluated using the Hosmer–Lemeshow goodness-of-fit test, as well as the Cox & Snell R² and Nagelkerke R². Results were expressed as exp(B). These were presented as odds ratios (ORs), with risk estimates reported as the OR alongside its 95% confidence interval (95% CI). In addition, ORs were adjusted for potential confounders (aORs), including CD duration, number of previous advanced treatments, previous surgery, and baseline C-reactive protein.

All analyses were conducted with a two-tailed significance level set at an alpha error of 5%, defining statistical significance as a p-value below 0.05. Statistical analyses were performed using IBM^®^ SPSS^®^ software (version 25 for Mac OS), while graphing was completed with Prism GraphPad^®^ (version 9 for Mac OS).

## Results

### Main characteristics of the study population

Of the 319 patients initially screened, 254 were eligible and included in the T_3_ analysis (the remainder were excluded because HBI could not be derived), the majority of whom were female (128, 50.4%), with median age and CD duration of 49 (39–62) and 12 (6–21) years, respectively.

Although the study also included UST for prophylactic purposes to prevent CD post-surgical recurrence, it should be emphasised that 89.7% of the patients included in the above-mentioned initial screening had an HBI of at least 5.

Clinical remission at T_3_ was observed in 30.7% of the sample. At 12 months (T4), the analysis focused on 143 patients with available data, slightly more often male (72, 50.3%), with a median age of 49 (39–60) years and a disease duration of 13 (8–21) years. Among these, 40.5% were in clinical remission at the T_4_ time point. Further detailed characteristics, stratified by remission status, are presented in Table 1. Moreover, a large proportion of the sample, both in the cohort selected for the T_3_ analysis and that for T_4_, had undergone previous surgery (54.7% and 60.1%, respectively).

**Table 1:** Univariate analysis of differences in clinico-demographic parameters collected at 6 months (T3) and 12 months (T4) from initiation of ustekinumab (UST) treatment between patients who had versus had not achieved clinical remission

Variable	6 Months (T_3_)				12 Months (T_4_)			
	Remission (*N*: 78)	No Remission (*N*: 176)	*p*-value ^1^	U/H/χ^2 3^	Remission (*N*: 58)	No Remission (*N*: 85)	*p*-value ^2^	U/H/χ^2 3^
Age	47.5 (39.75–57.25)	49.5 (38–62.75)	0.414	7305	51 (41–64)	48 (38–57)	0.082	2042
Males Females	37 (47.4%) 41 (52.6%)	89 (50.6%) 87 (49.4%)	0.645	0.212	27 (46.6%) 31 (53.4%)	45 (52.9%) 40 (47.1%)	0.453	0.563
Active smoker	11 (14.1%)	51 (29%)	0.104	6113.5	9 (15.5%)	21 (24.7%)	0.233	2.220
Appendectomy	18 (23.1%)	60 (34.1%)	0.079	3.081	13 (22.4%)	32 (37.6%)	0.054	3.710
IBD duration	13 (7.75–21.25)	12 (6–19)	0.415	6425.5	13.5 (8.75–21)	13 (6–22)	0.415	2267
A1, A2, A3	6 (7.7%), 58 (74.4%), 14 (17.9%)	22 (12.5%), 107 (60.8%), 47 (26.7%)	0.533	7148	4 (6.9%), 42 (72.4%), 12 (20.7%)	16 (18.8%), 53 (62.4%), 16 (18.8%)	0.174	2189
L1, L2, L3, L4	22 (28.2%), 16 (20.5%), 40 (51.3%), 0 (0%)	63 (35.8%), 32 (18.2%), 78 (44.3%), 3 (1.7%)	0.365	6411	16 (27.6%), 14 (24.1%), 28 (48.3%)	37 (43.5%), 15 (17.6%), 33 (38.8%)	0.100	2093
B1, B2, B3	33 (42.3%), 42 (53.8%), 3 (3.8%)	68 (38.6%), 81 (46%), 27 (15.3%)	0.154	7561	20 (34.5%), 32 (55.2%), 6 (10.3%)	27 (31.8%), 43 (50.6%), 15 (17.6%)	0.418	2643
Perianal disease	9 (11.5%)	20 (11.4%)	0.968	0.002	7 (12.1%)	13 (15.3%)	0.585	0.298
Previous surgery	40 (51.3%)	99 (56.3%)	0.463	0.538	31 (53.4%)	55 (64.7%)	0.224	1.823
Previous 5-ASA	44 (56.4%)	136 (77.3%)	**0.001**	11.394	40 (69%)	64 (75.3%)	0.404	0.696
Previous steroids	58 (74.4%)	145 (82.4%)	0.141	2.170	48 (82.8%)	66 (77.6%)	0.455	0.557
Previous AZA	40 (51.3%)	75 (42.6%)	0.369	1.993	33 (56.9%)	39 (45.8%)	0.334	2.195
Previous MTX	7 (9%)	24 (13.6%)	0.455	1.575	8 (13.8%)	11 (12.9%)	0.704	0.702
Previous GOL	1 (1.3%)	4 (2.3%)	0.600	0.275	0 (0%)	3 (3.5%)	0.272	2.091
Previous ADA	49 (62.8%)	117 (66.5%)	0.572	0.319	36 (62.1%)	62 (72.9%)	0.169	1.890
Previous IFX	47 (60.3%)	96 (54.5%)	0.397	0.716	33 (56.9%)	51 (60%)	0.711	0.137
Previous VDZ	17 (21.8%)	47 (26.7%)	0.406	0.691	11 (19%)	23 (27.1%)	0.264	1.246
N. of biologics	1 (43, 55.1%), 2(25, 32.1%), 3 (8, 10.3%)	1 (77, 43.8%), 2 (57, 32.4%), 3 (23, 13.1%), 4 (1, 0.6%)	0.726	7039	1 (26, 44.8%), 2 (18, 31%), 3 (6, 10.3%)	1 (33, 38.8%), 2 (30, 35.3%), 3 (14, 16.5%), 4 (1, 1.2%)	0.103	2839
Anti-TNF failure	74 (94.9%)	153 (86.9%)	0.058	3.587	50 (86.2%)	76 (89.4%)	0.561	0.338
Naïve	3 (3.8%)	18 (10.2%)	0.136	2.902	8 (13.8%)	7 (8.2%)	0.287	1.134
T_0_ ESR	(N:13): 43 (25–50)	(N:99): 35 (20–60)	0.598	585.5	(N:15): 28 (15–51)	(N:49): 30 (18–51.5)	0.887	358.5
T_0_ CRP	(N:70): 17 (8–22.25)	(N:157): 12 (5–24.5)	0.258	4978.5	(N:49): 17 (5–22.5)	(N:76): 8.85 (3–16.5)	**0.034**	1444
T_0_ FC	(N:52): 200 (138–246)	(N:129): 300 (210–806)	**< 0.0001**	4685	(N:42): 234 (184.25–500)	(N:59): 269 (195–1000)	0.166	1440
T0 SES-CD	(N:45): 11 (9–12)	(N: 105): 9 (8–13)	0.250	2084	(N:34): 10 (8–12)	(N:44): 9 (8–12.75)	0.487	679.5
T_0_ Rutgeerts	(N:26): i0 (3, 11.5%), i1 (3, 11.5%), i2 (4, 15.4%), i3 (13, 50%), i4 (3, 11.5%)	(N:70): i0 (2, 2.9%), i1 (9, 12.9%), i2 (19, 27.1%), i3 (16, 22.9%), i4 (24, 34.3%)	0.241	3532.5	(N:20): i1 (1, 5%), i2 (5, 25%), i3 (12, 60%), i4 (2, 10%)	(N:39): i1 (6, 15.4%), i2 (10, 25.6%), i3 (9, 23.1%), i4 (14, 35.9%)	0.713	412
T_1_ ESR	(N:16): 20 (12.5–29.5)	(N: 88): 28 (12–53.75)	0.114	4795.5	(N:13): 15 (10–23)	(N:45): 20 (11–45)	0.263	352.5
T_1_ CRP	(N:72): 5 (3–10)	(N: 143): 9 (3–20)	**0.017**	6167	(N:50): 8 (4–15)	(N:68): 6 (3–14)	0.521	1582.5
T_1_ FC	(N:19): 100 (40–200)	(N: 91): 253 (187–566)	**< 0.0001**	1339	(N:16): 282.5 (133.5–777.25)	(N:45): 250 (151–583)	0.718	338
T_1_ Steroids (yes)	18 (23.4%)	72 (43.1%)	**0.003**	8.819	20 (34.5%)	34 (44.2%)	0.256	1.290
T_2_ ESR	(N:14): 16.5 (7.25–27)	(N: 87): 19 (10–35)	0.330	4536	(N:18): 20 (13–28.5)	(N:46): 15 (10–33.25)	0.565	375.5
T_2_ CRP	(N:67): 3 (3–5)	(N: 141): 4.3 (2–10)	**0.011**	5743	(N:53): 3 (2.5–4.9)	(N:68): 3.25 (2–9)	0.255	2018
T_2_ FC	(N:19): 150 (89–400)	(N: 116): 178 (100–365)	0.697	1163.5	(N:21): 139 (58–495.5)	(N:56): 187.5 (113.75–392.5)	0.381	664.5
T_2_ Steroids (yes)	10 (13%)	55 (32.4%)	**0.001**	10.250	5 (8.6%)	35 (41.2%)	**0.000026**	17.707
T_3_ ESR	(N:14): 14 (8–20.5)	(N: 92): 18 (8–34.75)	0.255	5044	(N:14): 18.5 (11.5–36.25)	(N:50): 17.5 (8–33.5)	0.533	313.5
T_3_ CRP	(N: 66): 3 (2–4)	(N:151): 3.7 (3–13)	**0.002**	6257	(N: 53): 3 (2–5)	(N:73): 3 (2.75–14)	0.146	2222.5
T_3_ FC	(N:22): 181 (68.5–437)	(N: 116): 165 (88–354.5)	0.942	8074.5	(N:16): 282 (73.5–1517.25)	(N:57): 180 (98.5–400)	0.533	411.5
T_3_ Steroids (yes)	8 (10.5%)	42 (24.7%)	**0.011**	6.521	4 (6.9%)	26 (31%)	**0.001**	11.012
T_3_ SES-CD	(N:8): 4 (3.25–6.5)	(N: 52): 6 (4–9)	0.103	1660.5	(N:6): 6 (2.25–10)	(N:24): 6 (3.25–9.5)	0.938	73.5
T_3_ Rutgeerts	(N:11): 9 (81.8%), i1 (9.1%), i2 (9.1%)	(N:40): i0 (7, 17.5%), i1 (14, 35%), i2 (5, 12.5%), i3 (3, 7.5%), i4 (11, 27.5%)	**< 0.0001**	1191	(N: 7): i0 (3, 42.9%), i1 (1, 14.3%), i2 (2, 28.6%), i4 (1, 14.3%)	(N:21): i0 (1, 4.8%), i1 (9, 42.9%), i2 (3, 14.3%), i3 (2, 9.5%), i4 (6, 28.6%)	0.161	99
T_4_ ESR	N.A. ^2^	N.A. ^2^	N.A. ^2^	N.A. ^2^	(N:16): 14.5 (10–26.5)	(N:50): 19 (7.75–37.5)	0.736	422.5
T_4_ CRP	N.A. ^2^	N.A. ^2^	N.A. ^2^	N.A. ^2^	(N:52): 3 (2–3)	(N:72): 3 (1–12.52)	0.077	2214
T_4_ FC	N.A. ^2^	N.A. ^2^	N.A. ^2^	N.A. ^2^	(N:17): 198 (57.5–425)	(N:59): 175 (80–305)	0.789	523
T_4_ Steroids (yes)	N.A. ^2^	N.A. ^2^	N.A. ^2^	N.A. ^2^	2 (3.5%)	25 (29.4%)	**0.000320**	16.092
T_4_ SES-CD	N.A. ^2^	N.A. ^2^	N.A. ^2^	N.A. ^2^	(N:4): 7 (3.5–11.25)	(N:23): 5 (2–8)	0.513	36.5
T_4_ Rutgeerts	N.A. ^2^	N.A. ^2^	N.A. ^2^	N.A. ^2^	(N: 6): i0 (2, 33.3%), i2 (2, 33.3%), i4 (2, 33.3%)	(N:16): i0 (4, 25%), i1 (4, 25%), i2 (2, 12.5%), i3 (4, 25%), i4 (2, 12.5%)	0.763	44

Notes: Data are presented as median (interquartile range) or as frequency (percentage of the total), depending on whether variables were continuous or dichotomous/ordinal, with sample size (N) reported where applicable. The data derive from a retrospective, multicentre real-world study, with clinical remission defined as a Harvey–Bradshaw Index (HBI) < 5. CRP is expressed in mg/L, FC in µg/g, and ESR in mm/h

^1^ p-values were calculated using the Mann–Whitney U test, the Kruskal–Wallis test, or the Chi-square/Fisher’s exact test, as appropriate, with statistically significant values highlighted in bold

^2^ The analysis is not applicable (N.A.), as it refers to a timeframe beyond six months and is therefore not transferable with associative predictive value to a subsequent outcome setting

^3^ This column reports the numerical primary outcome of the test used, where applicable

*Acronyms*: A1–3, L1–4, B1–3 refer to the Montreal classification; IBD: inflammatory bowel disease; 5-ASA: 5-aminosalicylic acid derivatives; AZA: azathioprine; MTX: methotrexate; GOL: golimumab; ADA: adalimumab; IFX: infliximab; VDZ: vedolizumab; TNF: tumour necrosis factor; ESR: erythrocyte sedimentation rate; CRP: C-reactive protein; FC: faecal calprotectin; SES-CD: Simple Endoscopic Score for Crohn’s Disease

Given the higher patient drop-out at T_4_, Supplementary Table 1 shows a comparison between patients included in and excluded from this analysis with respect to the main variables related to disease phenotype, demonstrating substantial homogeneity of the sample with regard to most of these characteristics.

### Factors associated with lack of remission at six and twelve months

#### Baseline and medical history factors

As already highlighted in Table 1, in the univariate analysis, among the baseline clinical and demographic variables at T_3_, prior treatment with 5-ASA was more frequent in the non-remission group (136, 77.3%) than in the remission group (44, 56.4%; *p* = 0.001).

At T_3_, specific clinical and medical history variables were associated with the risk of not achieving remission. Previous exposure to 5-ASA [aOR: 2.704 (1.423–5.137), *p* = 0.002], a history of appendectomy [aOR: 1.965 (1.008–3.829), *p* = 0.047], and a diagnosis of CD before the age of 40 years [aOR: 2.03 (1.072–3.834), *p* = 0.04] were all associated with a higher risk of non-remission.

At T_4_, again a history of appendectomy remained a risk factor [aOR: 2.938 (1.212–7.124), *p* = 0.017].

#### Variables associated with disease activity trajectory

Parameters associated with disease activity showed distinct patterns of association with remission. In detail, as indicated in Table 2, overall, excluding the HBI across different time points (on which the model’s dependent variable was based), higher levels of CRP and FC at various time points were identified as risk factors for failure to achieve remission, whereas, conversely, the absence of steroid use at T_1_, T_2_, and T_3_ was associated with a reduced risk.

**Table 2:** Logistic regression analysis designed to identify variables associated with failure to achieve clinical remission at 6 and 12 months (T_3_ and T_4_, respectively) following initiation of ustekinumab (UST) treatment

Variable	6 Months (T_3_), *N*: 254			12 Months (T_4_), *N*: 143		
	aOR (95% CI)^1^	β	*p*-value	aOR (95% CI)	β	*p*-value
Previous 5-ASA	2.704 (1.423–5.137)	0.995	0.002	– ^2^	– ^2^	– ^2^
A2 (Montreal)	2.03 (1.072–3.834)	0.708	0.029	– ^2^	– ^2^	– ^2^
FC at T_0_^3^	1.22 (1.11–1.35)	0.002	0.006	– ^2^	– ^2^	– ^2^
Appendectomy	1.965 (1.008–3.829)	0.675	0.047	2.938 (1.212–7.124)	1.078	0.017
HBI at T_0_^4^	1.3 (1.156–1.462)	0.262	< 0.001	– ^2^	– ^2^	– ^2^
CRP at T_1_	1.073 (1.021–1.128)	0.07	0.006	– ^2^	– ^2^	– ^2^
FC at T_1_^3^	1.49 (1.11–2.22)	0.004	0.023	– ^2^	– ^2^	– ^2^
No steroids at T_1_	0.437 (0.233–0.822)	-0.827	0.01	– ^2^	– ^2^	– ^2^
CRP at T_2_	1.140 (1.05–1.238)	0.131	0.002	– ^2^	– ^2^	– ^2^
HBI at T_2_^4^	1.840 (1.515–2.234)	0.61	< 0.001	1.677 (1.364–2.061)	0.517	0.0001
No steroids at T_2_	0.313 (0.147–0.668)	-1.160	0.003	0.102 (0.033–0.314)	-2.283	< 0.001
HBI at T_3_^4^	N.A.	N.A.	N.A.	2.666 (1.839–3.864)	0.981	< 0.001
CRP at T_3_	1.138 (1.058–1.224)	0.129	0.001	1.1 (1.024–1.181)	0.095	0.009
No Steroids at T_3_	0.407 (0.176–0.943)	-0.898	0.036	0.173 (0.054–0.554)	-1.756	0.003
CRP at T_4_	N.A.	N.A.	N.A.	1.086 (1.01–1.168)	0.083	0.027
Remission at T_3_	N.A.	N.A.	N.A.	0.011 (0.002–0.055)	-4.528	< 0.001

Notes: For the sample size of each covariate variable, please refer to Table 1. The variables were considered at baseline (T_0_) and at 2 (T_1_), 3 (T_2_), 6 (T_3_), and 12 (T_4_) months following UST treatment. The data derive from a retrospective, multicentre real-world study, with clinical remission defined as a Harvey–Bradshaw Index (HBI) < 5. CRP is expressed in mg/L, FC in µg/g, and ESR in mm/h

^1^ The reported adjusted Odds Ratios (aORs) were estimated using multivariable logistic regression models for each exposure of interest, adjusted for a pre-specified set of potential confounders (i.e., CD duration, number of previous advanced treatments, previous surgery, and C-reactive protein at T_0_). For each model, the exposure of interest was excluded from the adjustment set to avoid conditioning on itself. The Hosmer-Lemeshow test was applied to assess model fit

^2^ If a dash (“–”) is present, this indicates that the comparison was not statistically significant. Conversely, “N.A.” suggests that the parameters cannot be logically included in the multivariate analysis, as they are not expected predictors

^3^ For FC, the OR is estimated for increments of 100 µg/g rather than for increments of 1 µg/g; therefore, the reported OR should be interpreted as OR_100_ = e^(β × 100)^

^4^ The HBI parameters, although collinear with the dependent variable (failure to achieve clinical remission at the considered time point), were included in the regression model to assess the model’s internal stability

*Acronyms*: 5-ASA: 5-aminosalicylic acid derivatives; CRP: C-reactive protein; FC: faecal calprotectin; HBI: Harvey-Bradshaw index

At this time point, disease activity variables and steroid use showed stronger associations, following the same pattern observed at T_3_ but in time periods closer to T_4_ (i.e., T_2_ and T_3_; see Table 2).

Interestingly, being in remission at T_3_ significantly increased the likelihood of remaining in remission at T_4_ [aOR: 0.011 (0.002–0.055), *p* < 0.001].

In the analysis of FC cut-offs, the one showing the greatest stability (both in terms of confidence interval and sample size in comparisons) was a baseline FC level (T_0_) ≥ 250, in relation to clinical remission at six months [aOR: 3.57 (1.686–7.561), *p* = 0.001]. Using a T_0_ FC cutoff of ≥ 250 µg/g, sensitivity for failure to achieve remission at T_3_ was 56.6% (95% CI 48.0–64.8%), with a specificity of 75.0% (95% CI 61.8–84.8%). The positive predictive value was 84.9% (95% CI 75.8–90.9%), whereas the negative predictive value was 41.1% (95% CI 31.7–51.1%).

### Variables correlated with disease activity at six and twelve months

As shown in Table 3, the HBI measured at 6 and 12 months of UST treatment showed several correlations with biochemical markers of disease activity. However, focusing on the six-month HBI, and considering only moderate-to-robust correlations while excluding weaker ones (which are detailed in Supplementary Figs. 1 and 2), these were observed with FC at T_0_ (ρ: 0.338, 95% CI 0.198–0.464, *p* < 0.001) and with HBI at T_0_ (ρ: 0.359, 95% CI 0.243–0.465, p 0.001), with a robust correlation with HBI at T_2_ (ρ: 0.757, 95% CI 0.695–0.807, *p* < 0.001). Conversely, a moderate correlation was found between the HBI measured at twelve months and the six-month HBI (ρ = 0.332, 95% CI 0.171–0.476, *p* < 0.001).

**Table 3:** Variables correlating with clinical disease activity, measured by the Harvey–Bradshaw Index (HBI), at both six months (T_3_) and twelve months (T4) of ustekinumab (UST) treatment

Variable	6 Months (T_3_), *N*: 254			12 Months (T_4_), *N*: 143		
	ρ ^1^	95% CI ^1^	*p*-value	ρ ^1^	95% CI ^1^	*p*-value
FC (T_0_)	**0.338**	**0.198–0.464**	**< 0.001**	– ^2^	– ^2^	– ^2^
HBI (T_0_)	**0.359**	**0.243–0.465**	**< 0.001**	– ^2^	– ^2^	– ^2^
ESR (T_1_)	0.222	0.025–0.402	0.023	– ^2^	– ^2^	– ^2^
CRP (T_1_)	0.200	0.064–0.328	0.003	– ^2^	– ^2^	– ^2^
FC (T_1_)	0.297	0.111–0.463	0.001	– ^2^	– ^2^	– ^2^
CRP (T_2_)	0.198	0.059–0.328	0.004	– ^2^	– ^2^	– ^2^
HBI (T_2_)	**0.757**	**0.695–0.807**	**< 0.001**	0.295	0.129–0.445	0.0004
CRP (T_3_)	0.199	0.063–0.326	0.003	– ^2^	– ^2^	– ^2^
FC (T_2_)	– ^2^	– ^2^	– ^2^	0.258	0.024–0.465	0.026
HBI (T_3_)	– ^2^	– ^2^	– ^2^	**0.332**	**0.171–0.476**	**< 0.001**

Notes: The data derive from a retrospective, multicentre real-world study, with clinical remission defined as a Harvey–Bradshaw Index (HBI) < 5. CRP is expressed in mg/L, FC in µg/g, and ESR in mm/h

^1^ According to Spearman’s analysis, Spearman’s coefficient (ρ) and relative 95% confidence interval (95% CI) values are interpreted as follows: > 0.69 (robust correlation), 0.40–0.69 (strong correlation), 0.30–0.39 (moderate correlation), 0.20–0.29 (weak correlation), 0.01–0.19 (no correlation). The variables were considered at baseline (T_0_) and at 2 (T_1_), 3 (T_2_), and 6 (T_3_) months following treatment. Moderate-to-robust correlation are indicated in bold for easy reference

^2^ If a dash (“–”) is present, this indicates that the comparison was not statistically significant

*Acronyms*: CD: Crohn’s disease; ESR: erythrocyte sedimentation rate; CRP: C-reactive protein; FC: faecal calprotectin

### Factors associated with lack of steroid-free remission at six and twelve months

#### Baseline and medical history factors

For this endpoint, data were available at T_3_ for 252 patients and at T_4_ for 89 patients, with steroid-free remission achieved in 27% and 38.2%, respectively.

For steroid-free clinical remission at T_3_, prior use of 5-ASA [aOR: 2.2 (1.124–4.306), *p* = 0.021], and a B2 phenotype [aOR: 2.271 (1.152–4.479), *p* = 0.018] were associated with a higher risk of not achieving remission. Conversely, for steroid-free remission at T_4_, a previous appendectomy emerged as a risk factor for failure to achieve this endpoint [aOR: 3.559 (1.430–8.858), *p* = 0.006].

#### Variables associated with disease activity trajectory

With respect to steroid-free remission at T_3_, as markers of disease activity, CRP at T_1_ [B: 0.102; aOR: 1.107 (1.042–1.177), *p* = 0.001] and CRP at T_2_ [B: 0.160; aOR: 1.173 (1.065–1.291), *p* = 0.001] were associated with a higher risk of not achieving remission. The same applied to a baseline FC ≥ 250 [aOR: 2.455 (1.145–5.264), *p* = 0.021]. The absence of steroids at T_1_ [aOR: 0.333 (0.166–0.669), *p* = 0.002] and at T_2_ [aOR: 0.048 (0.011–0.207), *p* < 0.001] was, as expected, associated with a protective effect.

As in the earlier analyses, for remission at T_4,_ the absence of steroids in the preceding periods was protective, specifically at T_2_ [aOR: 0.084 (0.026–0.276), *p* < 0.0001] and at T_3_ [aOR: 0.075 (0.017–0.343), *p* = 0.001]. As an internal control, having achieved steroid-free remission at six months was strongly associated with maintaining this status at twelve months [aOR: 0.009 (0.002–0.048), *p* < 0.0001].

## Discussion

Current literature data found that several characteristic of the patient undergoing to UST treatment may predict a positive or a negative response to the drug [11–13]. However, a source of bias could be the short-term analysis or the small sample size enrolled [11–13].

The data from this multicenter, real-life study, within the limitations inherent to its retrospective nature, confirmed that the treatment trajectory of patients with CD receiving UST to achieve clinically relevant endpoints depends on key variables. It is therefore advisable to ensure careful monitoring of biochemical markers of disease activity and to weigh the use of steroids judiciously, as their use may predict a subsequent poor response (i.e., a failure to achieve remission) to the drug.

Moreover, the population in which the analysis was conducted strongly reflects real-world clinical practice, as it consisted of patients with long-standing CD (with median disease durations well exceeding ten years), a heterogeneous disease phenotype, and a large majority who had already undergone surgery and had been previously exposed to another biological therapy, as evidenced by the low proportion of naïve patients included.

This directly aligns with the observed rates of clinical remission, recorded at 30.7% and 40.5% after six and twelve months of treatment, respectively. This latter finding, moreover, is consistent with what has already been observed in a previous Italian real-life experience conducted in the same setting and involving a sample with comparable characteristics (showing a clinical remission rate of 39% at 52 weeks) [20]. Furthermore, a recent meta-analysis of real-life studies reported similar pooled rates of clinical remission during UST maintenance therapy, amounting to 40% (95% CI: 28–54%; range: 26–73%; I² = 90%) [21].

One finding that emerged is that, aside from the biochemical and clinical variables associated with disease activity, specific anamnesis-related characteristics also showed a relevant associative weight. To begin with, prior treatment with 5-ASA increased the risk of failure to achieve clinical remission at six months (see Table 1), almost tripling the risk, and also steroid-free clinical remission at the same timepoint (aOR: 2.2). Indeed, this seems to support the clear advice that 5-ASA in CD has just an “historical” role [22–24], and should be no longer used in CD patient undergoing biologic therapy.

In addition, a history of appendectomy (Table 2) was another anamnesis-related factor associated with an increased risk of failing to achieve clinical remission at both six months (aOR: 1.965) and twelve months (aOR: 2.938). A similar pattern was observed for steroid-free remission. Although appendectomy is an established risk factor for the development of CD (weighted OR = 1.59 in a recent meta-analysis [25]), the impact of this surgical procedure on the clinical course of the disease has been less clearly investigated. Some studies, such as that by Riegler et al. [26], conducted on a small sample of forty CD patients, have shown that appendectomy prior to diagnosis was a risk factor for subsequent surgery. In addition, Cosnes et al. [27] showed that a prior appendectomy was associated (OR: 1.24) with an increased risk of intestinal stenosis. Conversely, another Danish population-based study on a large cohort suggested a milder disease course among those who had previously undergone appendectomy [28]. It is well known that the appendix functions as an immuno-microbial hub, a reservoir of commensal microbiota with biofilm for re-colonisation, possessing lymphoid structures that abundantly produce immunoglobulin A and natural killer T cells, as well as modulating T-helper inflammatory pathways [29]. Therefore, its removal may disrupt microbial homeostasis and mucosal immunity, with a potential impact on disease course, although the precise mechanisms remain to be elucidated in detail [29].

Finally, concluding the section on anamnesis-related variables, a diagnosis of CD before the age of forty (A2 according to the Montreal classification) was likewise associated with an increased risk (aOR: 2.03) of failure to achieve clinical remission at six months. Current literature data report conflicting results about the age at baseline as prognostic factor of response to UST [11–13, 30, 31], because several factors could be a source of bias in assessing this variable (such as perianal involvement, history of surgery, biologic-naïve status).

Beyond these factors, which are clearly non-modifiable and anamnesis-related (and should probably be regarded as potential predictors of reduced efficacy in populations similar to ours), the data from our study confirm the expected observation that patients presenting with a higher biochemical inflammatory burden have an increased risk of not responding to UST (as indicated by aORs > 1 for CRP and FC at T_0_ and T_1_ in relation to six-month remission, Table 2). The same applies to steroid use in the period preceding the six-month time point at which the endpoint is assessed. A similar pattern, as shown in the same table, was observed for the corresponding parameters evaluated closer to the twelve-month mark. It is noteworthy that steroid use, high disease activity, and elevated inflammatory markers are surrogate markers of disease severity [32], and this inevitably underscores the importance of closely monitoring such patients. When poor clinical response is accompanied by persistently altered laboratory parameters, the risk–benefit balance of waiting for a delayed remission that may never occur should be carefully reconsidered, as this could lead to further progression in disease severity and complications. All of this is further supported by the correlation analyses between clinical activity and inflammatory markers of disease, as shown in Table 3. Moreover, conversely, our data show that if a patient achieves clinical remission at six months of treatment, this is associated with a high likelihood of remaining in remission at twelve months (aOR 0.011), providing inverse evidence that when the drug proves effective at a reasonable interval after induction, there is a good probability of sustained treatment persistence, as also demonstrated in other real-life experiences [8, 33, 34].

In addition, another aspect highlighted by these data is the need for particular caution regarding the continued use of steroids alongside UST therapy, likely aimed at regaining response and/or promoting a delayed remission. As shown in Table 2, it is, on the contrary, the absence of steroids at T_1_, T_2_, and T_3_ following treatment that most strongly anticipates remission achievable at 6–12 months after UST induction. This suggests that if UST treatment requires the renewed use of steroids as early as two months after initiation, and subsequently thereafter, its anticipated effectiveness is likely to be limited.

Although dose-escalation regimens with UST (e.g. every four weeks) have been proposed and several experiences have suggested a potential benefit for patients [35], such approaches often remain difficult to implement across all healthcare settings because they may require off-label, ad hoc dosing practices, as is the case in Italy. Nonetheless, a maintenance regimen with an interval shorter than eight weeks remains categorically outside the drug’s approved label [15].

This study has several limitations. Its retrospective, multicentre design exposes it to information and selection biases; moreover, the real-world setting inevitably introduces missing data, which necessitated complete-case analyses that, in some analyses with smaller sample sizes, reduced statistical power. Aside from baseline variables, many associations between covariates and the endpoints are temporally proximate to the outcome, which entails a risk of reverse causation if interpreted in isolation. Instead, they should be understood as markers of disease activity and monitoring rather than independent causal effects in their own right. Endoscopic assessments are also limited, as is to be expected in a real-world rather than a randomised controlled trial design, and this clearly means that, consistent with the pre-specified endpoints, the study is primarily centred on the clinical aspect of remission. Certainly, the availability of a greater amount of endoscopic data in a larger proportion of patients would have strengthened the objective weight of the remission outcome, given that the HBI, although widely standardised in much of the literature on pharmacological response in Crohn’s disease, remains a criterion subject to a degree of subjectivity. The HBI was included in the regression models as a control covariate to assess the internal stability of the estimates with respect to clinical disease activity. Because the HBI is collinear and conceptually similar to the outcome, its inclusion may lead to over-adjustment. Accordingly, we prioritise the interpretation of the main models without HBI and regard those including HBI as sensitivity analyses.

Lastly, the association between steroid use at timepoints T_1_–T_3_ and failure to achieve clinical remission should certainly be interpreted with caution. In a real-world context, this relationship may very commonly be explained by confounding by indication and by temporal proximity to the endpoint, thereby framing steroid use as a possible marker of inadequate early disease control rather than as a true independent predictor of non-response to treatment.

In conclusion, these real-world data have suggested, in a population of CD patients largely post-surgical, complex, and bio-experienced, several associations between patient history variables (such as appendectomy, prior use of 5-ASA, and age at CD diagnosis) and response to UST treatment, reinforcing the importance of guidance by biochemical parameters and careful monitoring of steroid use.

## Supplementary Information

Below is the link to the electronic supplementary material.

Supplementary Material 1

Supplementary Material 2

Supplementary Material 3

## Data Availability

The datasets generated during and analysed during the current study are not publicly available but are available from the corresponding author on reasonable request.

## Abbreviations

5-ASA: 5-aminosalicylic acid
CD: Crohn’s disease
CI95%: Confidence interval 95%
CRP: C-Reactive protein
ESR: Erythrocyte sedimentation rate
FC: Faecal calprotectin
HBI: Harvey-bradshaw index
IL: Interleukin
SES-CD: Simple endoscopic score for crohn’s disease
UST: Ustekinumab
