# Supplementary material for: Analysis of possible baseline and treatment-course factors associated with non-remission in patients with Crohn’s disease treated with ustekinumab: a retrospective real-life analysis
Source: Pharmacol Rep. 2026 Mar 24;78(3):891–901. doi: 10.1007/s43440-026-00847-5 (PMC13275574; doi:10.1007/s43440-026-00847-5)
Supplement: Supplementary file 1 — Supplementary Material 1 [file 43440_2026_847_MOESM1_ESM.docx]

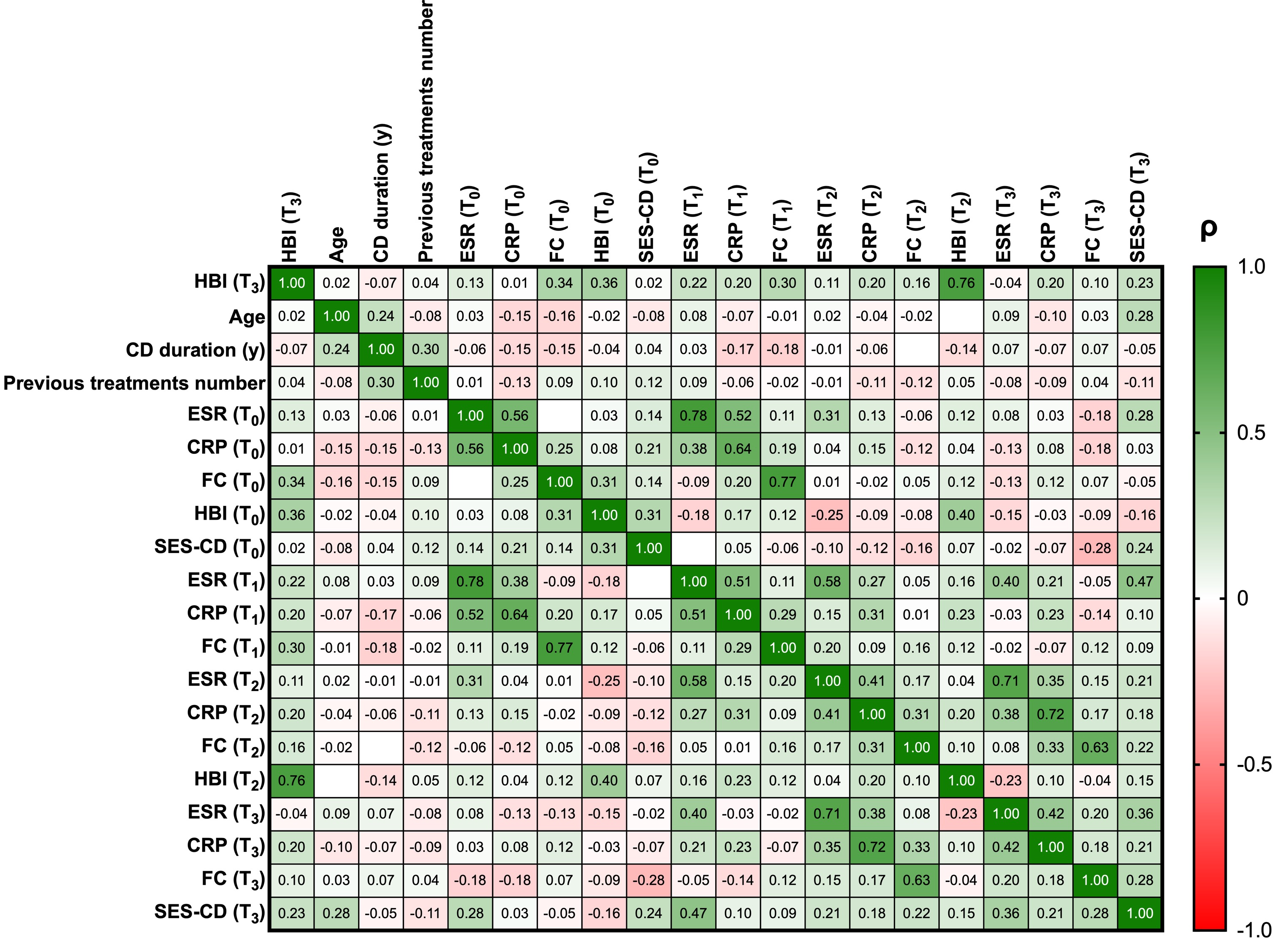


**Supplementary Figure 1.** Correlation matrix encompassing all continuous variables and specifically their relationship with disease activity measured by the Harvey–Bradshaw Index (HBI) at six months of ustekinumab (UST) treatment, on which the study’s primary endpoint is based. The variables were considered at baseline (T_0_) and at 2 (T_1_), 3 (T_2_), and 6 (T_3_) months following UST treatment start. The data derive from a retrospective, multicentre real-world study, with clinical remission defined as a Harvey–Bradshaw Index (HBI) < 5. CRP is expressed in mg/L, FC in µg/g, and ESR in mm/h.

According to Spearman’s analysis, Spearman’s coefficient (ρ) values are interpreted as follows: > 0.69 (robust correlation), 0.40–0.69 (strong correlation), 0.30–0.39 (moderate correlation), 0.20–0.29 (weak correlation), 0.01–0.19 (no correlation). In the graphical representation of the correlation matrix, a darker green or red colour corresponds to a stronger correlation.

*Acronyms*: CD: Crohn’s disease; ESR: erythrocyte sedimentation rate; CRP: C-reactive protein; FC: faecal calprotectin; SES-CD: Simple Endoscopic Score for Crohn’s Disease.
