# Supplementary material for: Analysis of possible baseline and treatment-course factors associated with non-remission in patients with Crohn’s disease treated with ustekinumab: a retrospective real-life analysis
Source: Pharmacol Rep. 2026 Mar 24;78(3):891–901. doi: 10.1007/s43440-026-00847-5 (PMC13275574; doi:10.1007/s43440-026-00847-5)
Supplement: Supplementary file 3 — Supplementary Material 3 [file 43440_2026_847_MOESM3_ESM.docx]

**Supplementary Table 1.** Table highlighting the main differences between the sample of patients with available data regarding the primary outcome of this study at 12 months (T_4_) and those not included.

| **Variable** | **T_4_ included population (N=143)** | **T_4_ excluded population (N=176)** | **p-value ^1^** | **U/H/χ^2^ ^2^** |
| --- | --- | --- | --- | --- |
| **Age** | 49 (39 – 61) | 49.5 (38 – 58.75) | 0.861 | 12727 |
| **Males** | 71 (49.7%) | 87 (49.4%) | 0.969 | 0.002 |
| **Number of previous treatments** | 1 (1 – 2) | 1 (1 – 2) | 0.824 | 12415 |
| **Active smoker** | 29 (20.3%) | 52 (29.5%) | 0.169 | 13565 |
| **Prior appendectomy** | 44 (30.8%) | 49 (27.8%) | 0.567 | 0.328 |
| **Montreal (A)**  A1  A2  A3 | 20 (14%)  94 (65.7%)  29 (20.3%) | 16 (9.1%)  119 (67.6%)  41 (23.3%) | 0.230 | 11766 |
| **Montreal (L)**  L1  L2  L3  L4 | 53 (37.1%)  29 (20.3%)  60 (42%)  1 (0.7%) | 44 (25%)  32 (18.2%)  98 (55.7%)  2 (1.1%) | **0.008** | 10581 |
| **Montreal (B)**  B1  B2  B3 | 46 (32.2%)  76 (53.1%)  21 (14.7%) | 84 (47.7%)  72 (40.9%)  20 (11.4%) | **0.009** | 14538 |
| **Montreal (p)** | 20 (14%) | 19 (10.8%) | 0.387 | 0.748 |
| **Previous surgery** | 84 (58.7%) | 88 (50%) | 0.119 | 2.426 |
| **Previous 5-ASA use** | 103 (72%) | 122 (69.3%) | 0.598 | 0.279 |
| **Previous steroid use** | 114 (79.7%) | 144 (81.8%) | 0.636 | 0.225 |
| **Previous thiopurine** | 70 (49%) | 75 (42.6%) | 0.266 | 2.648 |
| **Previous methotrexate** | 18 (12.6%) | 19 (10.8%) | 0.471 | 1.505 |
| **Previous GOL** | 3 (2.1%) | 4 (2.3%) | 0.916 | 0.011 |
| **Previous ADA** | 97 (67.8%) | 114 (64.8%) | 0.566 | 0.330 |
| **Previous IFX** | 83 (58%) | 106 (60.2%) | 0.693 | 0.156 |
| **Previous VDZ** | 33 (23.1%) | 46 (26.1%) | 0.529 | 0.396 |
| **T_0_ Rutgeerts**  i0  i1  i2  i3  i4 | N: 59  0 (0%)  7 (11.9%)  15 (25.4%)  21 (35.6%)  16 (27.1%) | N: 56  5 (8.9%)  6 (10.7%)  15 (26.8%)  17 (30.4%)  13 (23.2%) | 0.250 | 1850 |
| **ESR (T_0_)** | N: 64, 28.5 (16.5 – 50.75) | N: 82, 38.5 (26.5 – 60) | **0.009** | 1964 |
| **CRP (T_0_)** | N: 125, 12 (3.85 – 20.5) | N: 154, 12.5 (5.97 – 23.25) | 0.290 | 8916 |
| **FC (T_0_)** | N: 102, 235 (189.75 – 758.75) | N: 118, 251 (154 – 616.5) | 0.649 | 6232 |
| **SES-CD (T_0_)** | N:78, 9 (8 – 12) | N:102, 11 (8 – 14) | 0.122 | 3445 |

**Notes**: Data are presented as median (interquartile range) or as frequency (percentage of the total), depending on whether variables were continuous or dichotomous/ordinal, with sample size (N) reported where applicable. The data derive from a retrospective, multicentre real-world study, with clinical remission defined as a Harvey–Bradshaw Index (HBI) < 5. CRP is expressed in mg/L, FC in µg/g, and ESR in mm/h.

^1^ p-values were calculated using the Mann–Whitney U test, the Kruskal–Wallis test, or the Chi-square/Fisher’s exact test, as appropriate, with statistically significant values highlighted in bold.

^2^ This column reports the numerical primary outcome of the test used, where applicable.

*Acronyms*: A1–3, L1–4, B1–3 refer to the Montreal classification; 5-ASA: 5-aminosalicylic acid derivatives; AZA: azathioprine; MTX: methotrexate; GOL: golimumab; ADA: adalimumab; IFX: infliximab; VDZ: vedolizumab; TNF: tumour necrosis factor; ESR: erythrocyte sedimentation rate; CRP: C-reactive protein; FC: faecal calprotectin; SES-CD: Simple Endoscopic Score for Crohn’s Disease.
